# Malignancy-mimicking osteitis fibrosa cystica secondary to primary hyperparathyroidism

**DOI:** 10.1093/jscr/rjag749

**Published:** 2026-08-30

**Authors:** Shadi A Abu Isneina, Widad Abu Mayyala, Arein Madia, Abeer Madia, Majd Oweidat, Reham Hmedat, Jana Jawabra, Mohanad Jaber

**Affiliations:** College of Medicine and Health Sciences, Palestine Polytechnic University, Hebron, Palestine; Department of Orthopedic Surgery, Princess Alia Hebron Governmental Hospital, Hebron, Palestine; College of Medicine and Health Sciences, Palestine Polytechnic University, Hebron, Palestine; College of Medicine and Health Sciences, Palestine Polytechnic University, Hebron, Palestine; College of Medicine and Health Sciences, Palestine Polytechnic University, Hebron, Palestine; College of Medicine, Hebron University, Hebron, Palestine; College of Medicine and Health Sciences, Palestine Polytechnic University, Hebron, Palestine; College of Medicine and Health Sciences, Palestine Polytechnic University, Hebron, Palestine; College of Medicine and Health Sciences, Palestine Polytechnic University, Hebron, Palestine

**Keywords:** primary hyperparathyroidism, osteitis fibrosa cystica, brown tumour, parathyroid adenoma

## Abstract

Osteitis fibrosa cystica (OFC) is a rare skeletal complication of primary hyperparathyroidism (PHPT) that may present with multifocal osteolytic lesions mimicking multiple myeloma (MM) or metastatic disease. We report a 79-year-old woman with several years of generalized bone pain, fatigue, anorexia, and weight loss. Imaging revealed disseminated lytic lesions throughout the axial and appendicular skeleton, and MM was initially suspected. However, serum protein electrophoresis was normal, and bone marrow biopsy showed reactive changes with polyclonal plasma cells. She later developed a pathological midshaft femoral fracture. Laboratory testing showed severe hypercalcemia, markedly elevated parathyroid hormone, hypophosphatemia, elevated alkaline phosphatase, and vitamin D deficiency. Fracture fixation with targeted bone biopsy confirmed brown tumor consistent with OFC, and sestamibi scintigraphy localized a right inferior parathyroid adenoma. This case highlights that PHPT should be considered in patients with multifocal lytic bone lesions.

## Introduction

Primary hyperparathyroidism (PHPT) is an endocrine disorder caused by autonomous overproduction of parathyroid hormone (PTH), most commonly from a single parathyroid adenoma. Excess PTH disrupts calcium and phosphate homeostasis and promotes increased bone turnover, particularly through cortical bone resorption [1]. Although PHPT was historically recognized by the classic manifestations of ‘bones, stones, abdominal groans, and psychic moans’, its presentation has changed substantially with routine biochemical screening. Many patients are now diagnosed incidentally through asymptomatic hypercalcemia, whereas severe skeletal complications have become uncommon [2]. In ~85% of cases, PHPT is caused by a solitary adenoma, and severe skeletal disease may occur when hyperparathyroidism remains prolonged, untreated, or unrecognized [3].

Osteitis fibrosa cystica (OFC) is a late skeletal manifestation of sustained PTH excess and is characterized by generalized bone resorption, fibrous replacement of marrow, cystic bone change, and the formation of brown tumours [4]. Brown tumors are non-neoplastic osteolytic lesions composed of fibrovascular tissue, multinucleated giant cells, haemorrhage, and hemosiderin deposition [5]. They can involve the axial or appendicular skeleton and may present with bone pain, deformity, swelling, or pathological fracture [6].

This report describes a patient with severe PHPT presenting as malignancy-mimicking OFC with disseminated brown tumours and a pathological femoral fracture.

## Case presentation

A 79-year-old woman presented with a several-year history of generalized bone pain associated with fatigue, anorexia, and unintentional weight loss of 11 kg over 1 year. Her relevant medical history included hypertension and gout. No additional contributory social or family history was reported. Initial evaluation with whole-body computed tomography (CT) showed multiple bilateral eccentric cortical lytic lesions involving the femurs and patellae, with cortical disruption and soft-tissue extension. These findings raised concern for an underlying malignant bone process. However, the patient declined further evaluation at that time.

**Figure 1:**
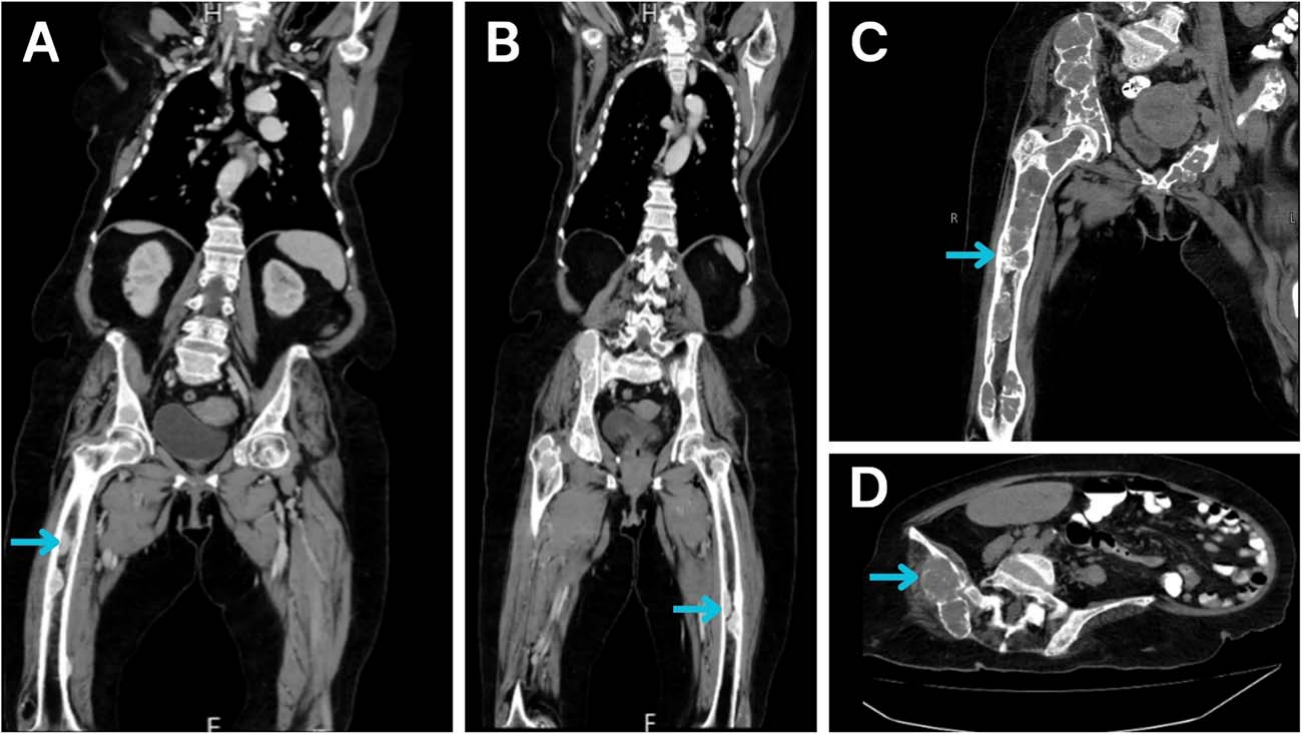
(A–C) Coronal views of post-contrast CT scan shows rounded cortically based lesions (arrows) in both femurs. (D) Axial view of CT scan without contrast of the pelvis shows expansile lesions (arrow) in the right iliac bone.

Two years later, the patient came to the department for further evaluation. A repeat whole-body CT showing disseminated osteolytic lesions throughout the axial and appendicular skeleton, including lesions in the ribs, humerus, glenoid cavities, iliac bones, and vertebrae (Fig. 1), described radiologically as hypermetabolic and suggestive of multiple myeloma (MM). No parathyroid mass was visualized on neck ultrasonography or CT. However, bone marrow biopsy showed reactive marrow changes with 8% polyclonal plasma cells rather than clonal plasma cell infiltration. In addition, serum plasma protein electrophoresis was normal. These findings did not support a diagnosis of MM.

**Figure 2:**
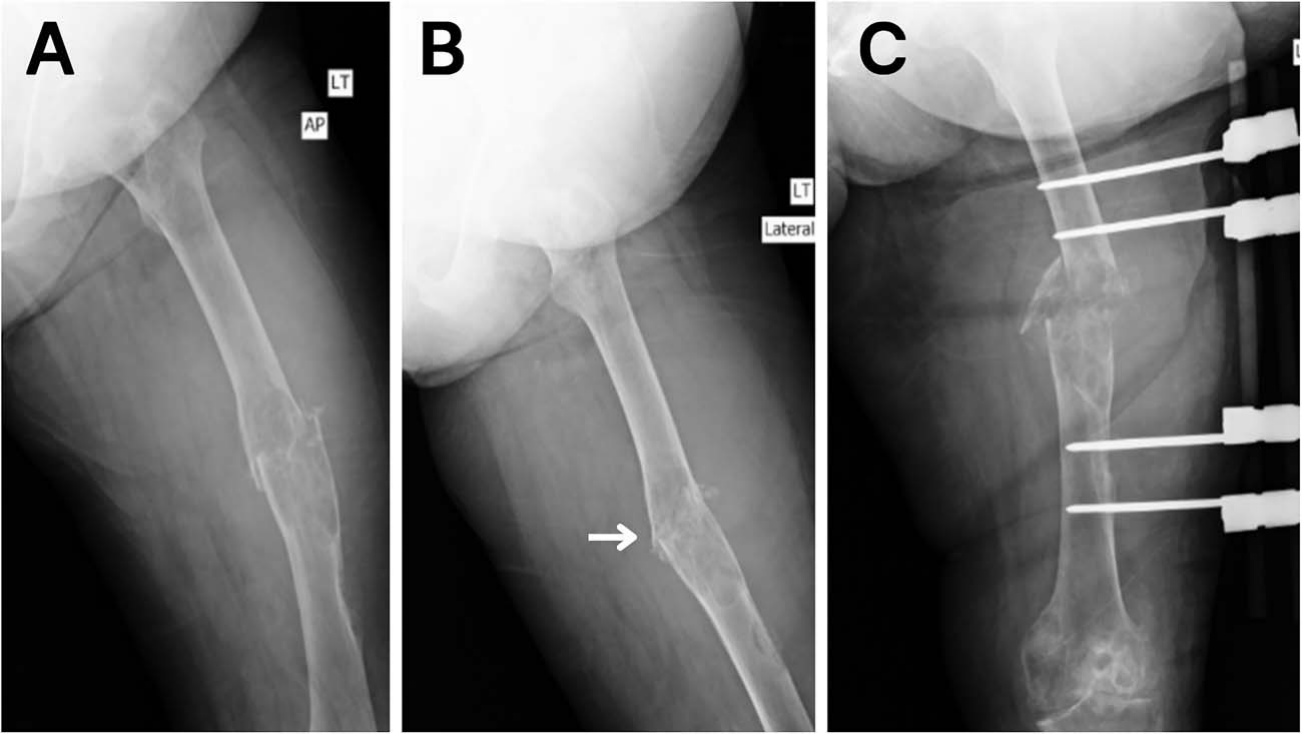
(A) AP view X-ray of the left femur. (B) AP view X-ray of the left femur, which shows a displaced fracture in the shaft of the femur (arrow). (C) AP view X-ray of the left femur shows the plates and screws used to fix the fracture.

One week later, the patient presented with acute left thigh pain. Plain radiographs confirmed a midshaft pathological fracture of the left femur (Fig. 2), requiring hospital admission. Laboratory investigations revealed markedly elevated PTH at 1654.1 pg/ml, and hypercalcemia with a serum calcium level of 14 mg/dl. Alkaline phosphatase (ALP) was elevated at 667 IU/L. The 25-hydroxyvitamin D level was low at 10.8 nmol/L. The chloride-to-phosphorus ratio was 62.78, calculated from chloride 113 and phosphorus 1.8, exceeding the diagnostic threshold of 33 and supporting PHPT. Serum electrolytes and kidney function tests were otherwise normal. Tumor markers, including carcinoembryonic antigen, cancer antigen (CA) 15–3, CA 19–9, and CA 125, were negative. Peripheral blood count showed mild anemia with haemoglobin of 8.5 g/dl and mean corpuscular volume of 70.7 fl, along with thrombocytosis of 600/μl.

**Figure 3:**
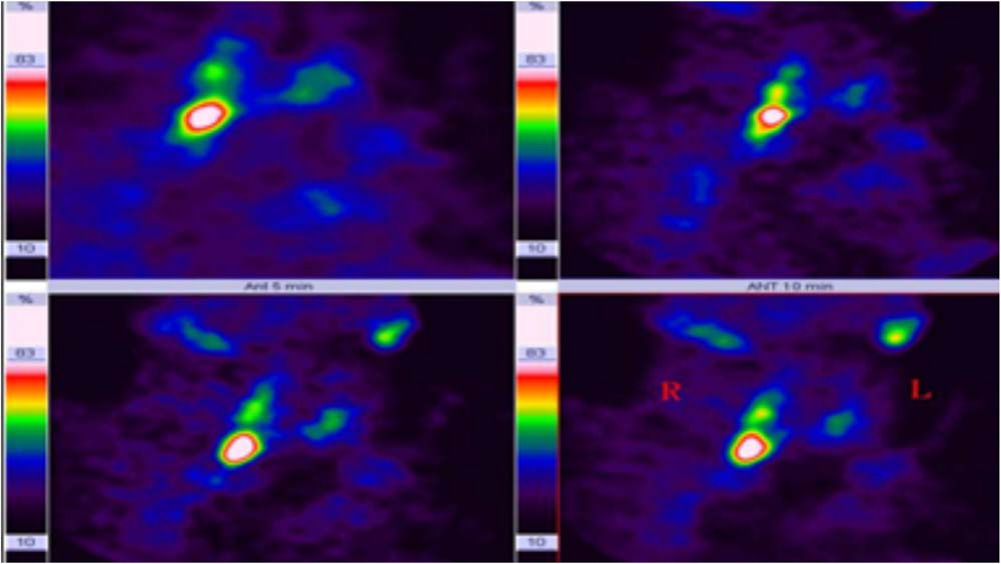
Parathyroid scan showed increased tracer uptake in the right lower pole of parathyroid glands.

The patient underwent open reduction and internal fixation of the left femoral pathological fracture (Fig. 2). An intraoperative targeted bone biopsy was obtained from the fracture site. Histopathological examination showed a pathological fracture with cortical thinning, replacement of bone marrow by fibrous tissue, and scattered multinucleated giant cells within a haemorrhagic fibroblastic stroma (brown tumour). These findings were consistent with OFC. No evidence of malignancy was identified. Subsequently, technetium-99 m sestamibi parathyroid scintigraphy showed a right inferior parathyroid adenoma (Fig. 3), confirming the source of autonomous PTH secretion.

Although parathyroidectomy was indicated, the patient declined surgery. Therefore, medical management was initiated with intravenous zoledronic acid, furosemide, cinacalcet 30 mg once daily, and alendronate 70 mg once weekly. The patient also continued her chronic medications for gout and hypertension. Planned surveillance included serial serum calcium, PTH, renal function, 25-hydroxyvitamin D, ALP, and orthopedic follow-up for fracture healing and functional recovery.

## Discussion

This case highlights an unusual presentation of PHPT in which advanced skeletal disease was initially interpreted as a possible malignant bone process.

Sustained PTH excess increases osteoblast expression of RANKL, which promotes osteoclast differentiation and activation. This leads to accelerated bone resorption, cortical thinning, cystic change, and skeletal fragility. Repeated microfracture and haemorrhage within resorbed bone produce brown tumours, which are not true neoplasms [7, 8]. In this patient, the same mechanism likely produced widespread cortical lytic lesions and ultimately a midshaft pathological femoral fracture.

A major point of interest is the diagnostic overlap between OFC and malignancy. Brown tumours can appear as solitary or multiple lytic lesions and may be hypermetabolic on imaging, which can mimic metastatic disease or MM [4]. Histology may also overlap with other giant-cell-rich lesions, including giant cell tumour and aneurysmal bone cyst [4]. Therefore, biochemical correlation is essential. In patients with osteolytic lesions and hypercalcemia, early measurement of PTH is a simple but important step.

Current management principles support parathyroidectomy as the definitive treatment for symptomatic PHPT and for PHPT complicated by skeletal disease [9]. This patient clearly met surgical criteria because of severe hypercalcemia and pathological fracture. Imaging should not be used to establish the diagnosis of PHPT; rather, it is used for preoperative localization after the biochemical diagnosis is made [10].

When parathyroidectomy is declined or contraindicated, medical therapy is supportive rather than curative. Hydration and loop diuretics after volume restoration may help manage severe hypercalcemia acutely, while intravenous bisphosphonates can reduce bone resorption temporarily [11]. Cinacalcet may reduce serum calcium by activating the calcium-sensing receptor, although it does not reliably improve bone mineral density. Bisphosphonates such as alendronate may improve bone mineral density but do not correct autonomous PTH secretion [1]. Therefore, the patient’s medical regimen should be interpreted as disease control rather than definitive cure.

In conclusion, in patients with malignancy-mimicking multifocal lytic bone lesions, clinicians should rule out PHPT and OFC.

## References

[1] Bilezikian JP, Bandeira L, Khan A et al. Hyperparathyroidism. Lancet 2018;391:168–78. 10.1016/S0140-6736(17)31430-7 28923463

[2] Insogna KL . Primary hyperparathyroidism. N Engl J Med 2018;379:1050–9. 10.1056/NEJMcp1714213 30207907

[3] Bilezikian JP, Khan AA, Silverberg SJ et al. Evaluation and management of primary hyperparathyroidism: summary statement and guidelines from the Fifth International Workshop. J Bone Miner Res 2020;37:2293–314. 10.1002/jbmr.4677 36245251

[4] Misiorowski W, Bilezikian JP. Osteitis fibrosa cystica. JBMR Plus 2020;4:e10403. 10.1002/jbm4.10403 32995697 PMC7507312

[5] Kalapala L, Keerthi Sai S, Babburi S et al. An endocrine jaw lesion: dentist perspective in diagnosis. Case Rep Dent 2016;2016:2582038. 10.1155/2016/2582038 27974979 PMC5126398

[6] Jervis L, James M, Howe W et al. Osteolytic lesions: osteitis fibrosa cystica in the setting of severe primary hyperparathyroidism. BMJ Case Rep 2017;2017:bcr-2017–220603. 10.1136/bcr-2017-220603 PMC5623214 28554885

[7] Sell J, Ramirez S, Partin M. Parathyroid disorders. Am Fam Physician 2022;105:289–98. 35289573

[8] Minisola S, Arnold A, Belaya Z et al. Epidemiology, pathophysiology, and genetics of primary hyperparathyroidism. J Bone Miner Res 2022;37:2315–29. 10.1002/jbmr.4665 36245271 PMC10092691

[9] Wilhelm SM, Wang TS, Ruan DT et al. The American Association of Endocrine Surgeons guidelines for definitive management of primary hyperparathyroidism. JAMA Surg 2016;151:959. 10.1001/jamasurg.2016.2310 27532368

[10] Yeh R, Tay Y-KD, Tabacco G et al. Diagnostic performance of 4D CT and Sestamibi SPECT/CT in localizing parathyroid adenomas in primary hyperparathyroidism. Radiology 2019;291:469–76. 10.1148/radiol.2019182122 30835187 PMC7440755

[11] KDIGO 2017 clinical practice guideline update for the diagnosis, evaluation, prevention, and treatment of chronic kidney disease–mineral and bone disorder (CKD-MBD). Kidney Int Suppl 2017;7:1–59. 10.1016/j.kisu.2017.04.001 PMC6340919 30675420

